# Place of residence & financial hardship: the situation of people with spinal cord injury

**DOI:** 10.1186/s12939-018-0818-9

**Published:** 2018-08-08

**Authors:** Diana Pacheco Barzallo

**Affiliations:** Swiss Paraplegic Research, Health Services Statistics & Economics Group, Guido A. Zach Strasse 4., 6207 Nottwil, Switzerland

**Keywords:** Financial hardship, Place of residence, Long-term care, Health system, Inequality

## Abstract

**Background:**

Even with universal health coverage, people with long-term medical conditions can face financial hardship. However, financial hardship can be not only the result of an increase in health care costs; it has other socio-economic determinants that can cause social inequalities in terms of health. This study aims to estimate the impact of the place of residence on the financial hardship of people with spinal cord injury (SCI) in Switzerland. Switzerland is an interesting case to analyze because of its political system, where each of the 26 cantons is autonomous and responsible for raising its own income (through taxes) and providing public services.

**Methods:**

Using cross-sectional data from the Swiss Spinal Cord Injury Cohort Study (SwiSCI), this paper estimates the probability of financial hardship by place of residence. The data set, recorded between 2011 and 2013, comprises information from 1549 participants aged 16 years and older, living with SCI.

**Results:**

The results show that people face different probabilities of financial hardship, depending on their place of residence. In general, people in the French-speaking cantons have a higher probability of financial hardship compared with people living in the German- or Italian-speaking cantons. People in the cantons of Geneva and Graubünden have almost five times the probability of financial hardship, compared with people in the canton with the lowest probability of financial hardship, Zug.

**Conclusions:**

The place of residence is a determinant of the financial situation of a household where a member deals with a long-term health condition. The differences might arise due to variations in health care costs, the tax burden and social support system, which are regulated and administered by each canton.

## Background

Long-term care can create catastrophic spending even for wealthy households [1]. Families with a member dealing with a long-term health condition have to finance a significant increase in their expenses that goes beyond the use of health care services. In many cases, financial hardship forces families to make difficult decisions and to choose between health care and basic living expenses; such a situation, in the long run, translates into poor health outcomes [2, 3] and in an increase in healthcare costs for the health system [4].

Financial hardship is determined by the socio-economic conditions of a person, such as education level, health status or working situation. At the same time, however, it could also be the result of indirect causes, such as the place of residence, where geographical differences can lead to inequalities in health [5]. For high-frequency users of the health system, for example, the prices of health services, the availability and accessibility of health providers, the support for care, and the socioeconomic environment can vary widely by geographic location, putting some families at a significant disadvantage.

Literature on neighborhood effects has long discussed how to operationalize, conceptualize and measure place effects [6]. In many cases, however, the inability to explain the causal link between health and place persists because it is difficult to justify geographical areas for comparison [7, 8]. This is because health is not only determined by personal factors, but also by social and environmental factors that are not always directly observed and, therefore, difficult to quantify [9]. Also, boundaries between neighborhoods are not always obvious and depend on many factors, where spatial contexts are more likely to have a fuzzy effect [9]. For example, subjects near the boundaries might be influenced by the characteristics of more than one place. Finally, the effect within regions might not be the same for all residents, where intra-correlations could vary depending on how the neighborhood is defined [5]. While smaller spaces are important for social interactions, larger areas can capture the socioeconomic conditions of the neighborhood.

The aim of this study is to show that high-frequency users of health services face an adverse financial situation due to their place of residence. I use a large and comprehensive data set that reports the living conditions of people with Spinal Cord Injury (SCI) in Switzerland. People with SCI are of special interest to the health system because their situation is a good reflection of the population’s health in the near future. Ageing together with the prevalence of chronic illnesses predict that most people, at some point in their life time, will have to deal with some kind of disability— either of their own or that of a family member [10].

Switzerland is a unique case for the analysis of place and health because of its decentralized political system. Although the health system functions under universal coverage, each of the 26 cantons is autonomous and responsible for funding and hospital planning, where healthcare subsidies play an important role in reducing the burden on low-income households [11, 12]. Health insurance is mandatory for all residents and the premiums are set by the cantons (community rating) and can only vary by age, level of deductible, and supplementary insurance plans. The costs of health services are set by the market, but controlled at the federal level and the cantonal administrations have the right to approve them. On average, households experience an out-of-pocket expenditure close to 26% of total health spending, which is significantly higher compared with other OECD countries [13].

Empirical evidence has shown significant geographical variation in Switzerland by language region in terms of cost of care. These differences are associated with cultural factors that affect the delivery and utilization of health services [14]. During the last years of life, for example, people in the French- and Italian-speaking regions are more likely to die in a hospital than at home or in a nursing home. Costs in hospitals are higher, which could partly explain the differences among regions. Similar variations occur with doctors’ decisions; doctors in the French-speaking region tend to treat pain and symptoms more aggressively than doctors in the German- and Italian-speaking regions [15]. Significant differences also exist between rural and urban areas; consultation costs are significantly higher in the latter. The difference can be explained by people’s preferences to seek more specialized care in urban areas [16].

At the cantonal level, previous literature has found significant variations in per capita health expenditure, which can be related to the density of physicians and to demographic characteristics of the cantons [17]. Health infrastructure could also explain some differences among cantons, where people living in smaller or more rural cantons could be at a disadvantage [13, 18]. Nevertheless, among OECD countries, Switzerland has one of the highest numbers of hospitals relative to the population. There are 45 rehabilitation hospitals; four are specialized SCI centers and 10 are acute hospitals which specialize in spine and neurosurgery [19, 20]. Patients are free to move between cantons to access health services, which reduces the potential inequalities that could arise and availability of the services in each location. Estimates show that, on average, 99% of the population can reach a hospital in less than 20 min [21].

Finally, a great part of the variation in equality among cantons can be rooted in the taxation system [22]. Existing evidence shows that the system is regressive, thus impacting the health system [23]. In Switzerland, households are subject to tax liability defined by the canton of residence, where the tax rate varies depending on the household size, working status and income level. This structure has raised important differences and similar households face different levels of tax burden [24, 25]. In general, married couples where both partners work have a higher tax burden compared with single person households or households with children.

## Methods

### Sample

This paper employs the Swiss Spinal Cord Injury Cohort Study (SwiSCI). SwiSCI is a cross-section study of people with SCI (traumatic and non-traumatic), which includes information from 1549 participants, aged 16 years and older, living in Switzerland between the years 2011 and 2013.

SwiSCI is the largest national cohort study about persons with SCI in Europe and it evaluates functioning, disability and health [26]. The survey was conducted according to the ethical principles formulated in the Declaration of Helsinki and the study protocol was approved by the Medical Ethics Committee of the Canton of Lucerne and by the Ethics Committees of all Swiss cantons involved.

The study population was established from the records of three specialized rehabilitation centers and two national associations of people with SCI. The eligible population included 3144 persons. Subjects had the option to complete the questionnaire using a paper and pencil version or online format; for those not willing or unable to use either of these two options, a telephone interview was conducted. Nearly 50% of the eligible subjects completed all modules of the questionnaire and non-response bias was found to be a minor issue [26].

### Statistical analysis

The questionnaire asked participants to rate their levels of financial hardship using a 4-point scale: “not applicable,” “no influence,” “a little harder” and “a lot harder.” Due to a small number of participants answering “a lot harder”, instead of implementing an ordered model, a binary model was implemented to estimate the probability of financial hardship by canton of residence.

The variable ‘financial hardship’ was recoded into a binary outcome that grouped respondents into two groups: people who reported no financial hardship (69.7%; those who answered “not applicable” and “no influence”) and people who reported suffering from some financial hardship (30.3%; those who answered “a little harder” and “a lot harder”).

The equation that estimated the probability of financial hardship by the canton of residence is shown in Eq. 1. For ease of interpretation, the equation is written in linear form:

$$ {y}_i=\propto +\sum \limits_{k=1}^{19}{\gamma}_k{z}_k+\sum \limits_j{\beta}_j{x}_{ij}+{\varepsilon}_i $$

where *y*_*i*_ is equal to 1 if the person reported financial hardship, and 0 otherwise. The vector *x*_*ij*_ controls for socio-demographic characteristics of individual *i* living in the household *j*, which have been identified in the literature to have an effect on the financial situation of a person. Among these variables, individuals and household characteristics were included. The variables included were sex, age, age^2^, nationality, health quality, SCI type, SCI cause, partnership situation, children in the household, education level, occupational situation, household income, language of the region, and spoken language. A detailed description of each variable is reported in Table 1.

**Table 1:** Variables included in the regression analysis

Variable	Type	Description
sex	binary	1 = male; 0 = otherwise
age	continous	
age^2^	continous	
nationality	binary	1 = Swiss; 0 = otherwise
has a partner	binary	1 = married or in a relationship; 0 = otherwise
children in the household	binary	1 = children live in the household; 0 = otherwise
SCI type	binary	1 = paraplegia; 0 = tetraplegia
SCI cause	binary	1 = traumatic; 0 = nontraumatic
health quality	categorical	1 = very dissatisfied; 2 = dissatisfied; 3 = neither satisfied, nor dissatisfied; 4 = satisfied; 5 = very satisfied
Spoken language:		
French	binary	1 = speaks French; 0 = speaks German
Italian	binary	1 = speaks Italian; 0 = speaks German
Language region:		
French-speaking canton	binary	1 = French-speaking; 0 = German-speaking
Italian-speaking canton	binary	1 = Italian-speaking; 0 = German-speaking
Education:		
vocational	binary	1 = vocational education; 0 = compulsory education
secondary	binary	1 = secondary education; 0 = compulsory education
university	binary	1 = university education; 0 = compulsory education
Occupational situation:		
full-time job	binary	1 = has a full-time payed activity; 0 = otherwise
part-time job	binary	1 = has a part-time payed activity; 0 = otherwise
in education	binary	1 = is in education; 0 = otherwise
unpaid work	binary	1 = non payed activity; 0 = otherwise
unemployed	binary	1 = searching for a job; 0 = otherwise
homemaker	binary	1 = stays at home; 0 = is unemployed
invalidity pension	binary	1 = receives invalidity pension; 0 = iotherwise
retired	binary	1 = is retired; 0 = otherwise
other activity	binary	1 = additional studies, voluntary activities, etc.; 0 = otherwise
household income	categorical	1 = < 1500 CHF; 2 = 1500–3000 CHF; 3 = 3000–4500 CHF; 4 = 4500–6000 CHF; 5 = 6000–7500 CHF; 6 = 7500–9000 CHF; 7= > 9000 CHF
Cantonal variables:	binary	19 dummy variables were included. 1 = selected canton; 0 = canton of Zug

The variable ‘sex’ controls for the gender gap. Age and age^2^ account for the non-linear evolution of the financial situation of a person over time, because younger and older people tend to earn less. Nationality is important in the Swiss context, since many studies have shown that, depending on the migration background, people face different opportunities in the labor market [22].

To control for the health conditions of a person, the regression included three variables: First, the health quality, which is a self-perceived measure of health and is proven to be a good predictor of the use of health services [27]. Second is the type of SCI, a variable that groups people into paraplegic and tetraplegia categories. This variable controls for the extent of the injury, which reflects the potential adaptability of a person to the labor market. Third is the cause of SCI, which shows whether the injury was caused by a traumatic or a non-traumatic event. People in the traumatic group are covered by accident insurance and not by regular health insurance. In general, accident insurance includes a more generous package and patients have to pay a lesser deductible for the treatments.

In terms of family composition, if an individual has a partner or family dependents (individuals younger than 16 years old), this significantly affects the family budget. On the one hand, people living in partnerships are more likely to have a higher income because there is more than one person able to work in the household. On the other hand, children in the household imply more expenses and, maybe, less time availability for the adults to work.

In the Swiss context, it is important to differentiate between the language of the region and the spoken language because these are not always the same. While the language region controls for geographical characteristics, the spoken language controls for the population’s linguistic abilities. Switzerland has four official languages: German, French, Italian and Romansh. Most cantons have one official language and, at school, people learn a second official language. In a multilingual country, where near two-thirds of the population speaks at least two languages [28], mastering an additional language is very important in the labor market.

Education, occupational situation and income level control for the potential opportunities individuals face due to their skills (human capital) and economic conditions. The household income reflects the flexibility of the finances of a family. In general, families with higher incomes will have less pressure if one of the members requires long-term care and/or is unable to work.

The place of residence was included in the regression using 19 dummy variables (z_k_), where a variable was equal to 1 if a person lived in a given canton and equal to 0 for the canton of reference. For comparison purposes, the canton of reference was Zug because it showed the lowest financial hardship in the sample.

Even though there might be intra-cantonal correlation, it is not possible to cluster the error term (*ε*_*i*_) at cantonal level because the number of clusters is small. Since the equation is a non-linear model, the use of bootstrapping techniques is not feasible either. Nevertheless, the large sample size and the cantonal fixed effects included in the regression reduce, to a great extent, the potential bias in the results.

A potential problem in the estimation is the endogeneity of the ‘health status’ variable included in the covariates. In fact, there exists an extensive body of literature analyzing financial constraints as a risk factor for obesity, low birth-weight and mental health, among other health conditions [25, 29–31]. However, SCI is mostly caused by a traumatic event. Therefore, we will only consider financial hardship as a result of a health condition and not the other way around. If there exists an effect of financial hardship on health status, it is more likely linked to cantonal characteristics. If this is the case, the cantonal fixed effects will absorb this effect.

### Robustness check

To test the robustness of the estimates, the statistical analysis replicated Eq. (1) using the same covariates, but different versions of the place of residence. The alternative versions of Eq. (1) included the language region and the statistical region (Nomenclature of Territorial Units for Statistics (NUTS)).

## Results

### Descriptive statistics

Table 2 displays the main characteristics of the SwiSCI sample. As described in the previous section, the variable ‘financial hardship’ was recoded to classify the sample into two groups: people who reported some level of financial hardship (69.7%) and people who reported having no financial hardship (30.3%).

**Table 2:** Descriptive Statistics SwiSCI Data

Variable	N	(%)
male	1107	71.5%
age in years	52.3 (mean)	
have a partner	1004	67.5%
have children	300	19.5%
nationality: Swiss	1363	91.3%
Language		
German	1088	70.2%
French	391	25.2%
Italian	70	4.5%
Educational attainment		
compulsory education	143	9.4%
vocational training	377	24.9%
secondary education	721	47.5%
university	276	18.2%
Occupational situation		
in work	684	43.3%
education	63	4.0%
unemployed	41	2.6%
retired	380	24.0%
Income level		
< 1500 CHF	47	3.2%
1500–3000 CHF	156	10.7%
3000–4500 CHF	289	19.9%
4500–6000 CHF	356	24.5%
6000–7500 CHF	230	15.8%
7500–9000 CHF	189	13.0%
> 9000 CHF	187	12.9%
no financial hardship	1031	69.7%
financial hardship	448	30.3%
Health quality		
very dissatisfied	100	6.6%
dissatisfied	270	18.0%
neither satisfied nor dissatisfied	378	25.1%
satisfied	634	42.2%
very satisfied	122	8.1%
Injury characteristics		
traumatic SCI	1202	78.4%
person with paraplegia	1063	69.2%
Observations	1549	100%

In terms of socio-economic characteristics, on average, participants were 52-year-old females from Switzerland. Among the sample, 67.5% reported having a partner and not many reported living with relatives younger than 16 years. A great portion of the sample spoke German (70.2%), a quarter spoke French, and only 4.5% spoke Italian. More than the 70% of the respondents had secondary education or vocational training, and 18.2% had a university degree.

In terms of working conditions, almost half of the sample was engaged in productive activity (43.3%), while 24% were already retired. There were a small number of people who were unemployed (2.6%), while 4% were still pursuing some type of education. Sixty percent of the sample had a monthly income between CHF 3000 and CHF 7500; 3% earned below CHF 1500 and 13% earned above CHF 9000.

50.3% of the sample reported being satisfied with their health, only 24.6% reported some level of dissatisfaction. In terms of injury characteristics, 70% had paraplegia and close to 80% reported having SCI due to a traumatic event.

For each respondent to the survey, we recorded their canton of residence. Nevertheless, due to privacy issues, cantons with less than 15 participants were not included in the analysis. This exclusion reduced the sample to 1240 participants in 19 cantons.

### Regression analysis

The results of Eq.(1) are reported in Table 3. For ease of interpretation, the table reports the marginal effects of the regression and not the estimated coefficients. The marginal effects can be read as the change in the probability of financial hardship, given a marginal change in the covariates. For the dummy variables, however, the marginal results have to be interpreted with respect to the reference group, which for the cantonal variables is the canton of Zug. In the Appendix, Table 4 compares the probability of financial hardship across cantons.

**Table 3:** Results: Logit Regression

	Marginal effects	Standard errors
Personal / family characteristics:		
sex: male	0.050	(0.034)
age	0.016*	(0.007)
age^b^	0.000*	(0.000)
nationality: Swiss	−0.035	(0.055)
health quality	−0.078***	(0.015)
SCI type: paraplegia	0.017	(0.031)
SCI cause: traumatic	−0.094**	(0.035)
has a partner	0.042	(0.032)
children in the household	0.085*	(0.037)
Spoken language:^a^		
German	0.071	(0.113)
French	−0.058	(0.117)
Education:^b^		
vocational	0.016	(0.057)
secondary	−0.011	(0.056)
university	0.070	(0.064)
Occupational situation:		
full-time job	0.076	(0.090)
part-time job	0.120	(0.091)
in education	0.010	(0.078)
unpaid work	0.023	(0.090)
unemployed	0.200^*^	(0.079)
homemaker	−0.073	(0.044)
invalidity pension	−0.028	(0.090)
retired	−0.026	(0.054)
other activity	0.152^*^	(0.065)
household income	−0.110^***^	(0.010)
Canton of residence:^c^		
AG: Aargau	0.213	(0.158)
BE: Bern	0.277	(0.156)
BL: Basel-Landschaft	0.191	(0.161)
BS: Basel-Stadt	0.139	(0.175)
FR: Fribourg	0.208	(0.175)
GE: Geneva	0.385^*^	(0.182)
GR: Graubunden	0.386^*^	(0.171)
JU: Jura	0.216	(0.203)
LU: Lucerne	0.272	(0.159)
NE: Neuchatel	0.364	(0.192)
SG: St. Gallen	0.194	(0.165)
SO: Solothurn	0.155	(0.171)
SZ: Schwyz	0.291	(0.179)
TG: Thurgau	0.329^*^	(0.164)
TI: Ticino	0.243	(0.196)
VD: Vaud	0.343^*^	(0.170)
VS: Valais	0.320	(0.166)
ZH: Zurich	0.241	(0.156)
Observations	1233	

Robust standard errors in parenthesis

For discrete variables, the marginal effects should be interpreted respect to the reference group:

^a^Italian is the reference group

^b^Compulsory education is the reference group

^c^The canton of Zug is the reference group

*p < 0.05, **p < 0.01, ***p < 0.001

**Table 4:** Financial Hardship: Cross-Cantonal Marginal Effects

	ZH	ZG	VS	VD	TI	TG	SZ	SO	SG	NE	LU	JU	GR	GE	FR	BS	BL	BE	AG
AG: Aargau	-0.03 (-0.45)	0.21 (1.34)	-0.11 (-1.35)	-0.13 (-1.47)	-0.03 (-0.23)	-0.12 (-1.48)	-0.08 (-0.75)	0.06 (0.63)	0.02 (0.25)	-0.15 (-1.19)	-0.06 (-0.90)	-0.00 (-0.02)	-0.17 (-1.92)	-0.17 (-1.58)	0.00 (0.05)	0.07 (0.72)	0.02 (0.32)	-0.06 (-1.10)	
BE: Bern	0.04 (0.67)	0.28 (1.77)	-0.04 (-0.59)	-0.07 (-0.83)	0.03 (0.27)	-0.05 (-0.70)	-0.01 (-0.14)	0.12 (1.38)	0.08 (1.16)	-0.09 (-0.72)	0.00 (0.08)	0.06 (0.45)	-0.11 (-1.28)	-0.11 (-1.04)	0.07 (0.78)	0.14 (1.40)	0.09 (1.33)		0.06 (1.10)
BL: Basel-Landschaft	-0.05 (-0.75)	0.19 (1.19)	-0.13 (-1.50)	-0.15 (-1.62)	-0.05 (-0.38)	-0.14 (-1.66)	-0.10 (-0.94)	0.04 (0.37)	-0.00 (-0.04)	-0.17 (-1.31)	-0.08 (-1.14)	-0.03 (-0.17)	-0.20* (-2.03)	-0.19 (-1.70)	-0.02 (-0.18)	0.05 (0.49)		-0.09 (-1.33)	-0.02 (-0.32)
BS: Basel-Stadt	-0.10 (-1.03)	0.14 (0.79)	-0.18 (-1.62)	-0.20 (-1.75)	-0.10 (-0.67)	-0.19 (-1.72)	-0.15 (-1.17)	-0.02 (-0.13)	-0.05 (-0.49)	-0.22 (-1.53)	-0.13 (-1.30)	-0.08 (-0.48)	-0.25* (-2.05)	-0.25 (-1.84)	-0.07 (-0.56)		-0.05 (-0.49)	-0.14 (-1.40)	-0.07 (-0.72)
FR: Fribourg	-0.03 (-0.34)	0.21 (1.19)	-0.11 (-1.37)	-0.13 (-1.71)	-0.03 (-0.24)	-0.12 (-1.15)	-0.08 (-0.66)	0.05 (0.46)	0.01 (0.14)	-0.16 (-1.29)	-0.06 (-0.67)	-0.01 (-0.06)	-0.18 (-1.60)	-0.18 (-1.69)		0.07 (0.56)	0.02 (0.18)	-0.07 (-0.78)	-0.00 (-0.05)
GE: Geneve	0.14 (1.34)	0.38* (2.12)	0.06 (0.67)	0.04 (0.45)	0.14 (0.96)	0.06 (0.47)	0.09 (0.68)	0.23 (1.79)	0.19 (1.62)	0.02 (0.16)	0.11 (1.02)	0.17 (1.19)	-0.00 (-0.01)		0.18 (1.69)	0.25 (1.84)	0.19 (1.70)	0.11 (1.04)	0.17 (1.58)
GR: Graubunden	0.15 (1.65)	0.39* (2.26)	0.07 (0.67)	0.04 (0.41)	0.14 (1.10)	0.06 (0.56)	0.09 (0.77)	0.23* (2.07)	0.19 (1.94)	0.02 (0.16)	0.11 (1.26)	0.17 (1.12)		0.00 (0.01)	0.18 (1.60)	0.25* (2.05)	0.20* (2.03)	0.11 (1.28)	0.17 (1.92)
JU: Jura	-0.02 (-0.18)	0.22 (1.06)	-0.10 (-0.81)	-0.13 (-1.01)	-0.03 (-0.16)	-0.11 (-0.77)	-0.08 (-0.46)	0.06 (0.39)	0.02 (0.15)	-0.15 (-0.96)	-0.06 (-0.40)		-0.17 (-1.12)	-0.17 (-1.19)	0.01 (0.06)	0.08 (0.48)	0.03 (0.17)	-0.06 (-0.45)	0.00 (0.02)
LU: Lucerne	0.03 (0.51)	0.27 (1.72)	-0.05 (-0.59)	-0.07 (-0.79)	0.03 (0.22)	-0.06 (-0.72)	-0.02 (-0.18)	0.12 (1.26)	0.08 (1.00)	-0.09 (-0.72)		0.06 (0.40)	-0.11 (-1.26)	-0.11 (-1.02)	0.06 (0.67)	0.13 (1.30)	0.08 (1.14)	-0.00 (-0.08)	0.06 (0.90)
NE: Neuchatel	0.12 (0.98)	0.36 (1.89)	0.04 (0.39)	0.02 (0.19)	0.12 (0.74)	0.03 (0.25)	0.07 (0.48)	0.21 (1.47)	0.17 (1.27)		0.09 (0.72)	0.15 (0.96)	-0.02 (-0.16)	-0.02 (-0.16)	0.16 (1.29)	0.22 (1.53)	0.17 (1.31)	0.09 (0.72)	0.15 (1.19)
SG: St. Gallen	-0.05 (-0.62)	0.19 (1.17)	-0.13 (-1.39)	-0.15 (-1.51)	-0.05 (-0.36)	-0.14 (-1.52)	-0.10 (-0.86)	0.04 (0.38)		-0.17 (-1.27)	-0.08 (-1.00)	-0.02 (-0.15)	-0.19 (-1.94)	-0.19 (-1.62)	-0.01 (-0.14)	0.05 (0.49)	0.00 (0.04)	-0.08 (-1.16)	-0.02 (-0.25)
SO: Solothurn	-0.09 (-0.95)	0.15 (0.91)	-0.16 (-1.60)	-0.19 (-1.70)	-0.09 (-0.59)	-0.17 (-1.69)	-0.14 (-1.09)		-0.04 (-0.38)	-0.21 (-1.47)	-0.12 (-1.26)	-0.06 (-0.39)	-0.23* (-2.07)	-0.23 (-1.79)	-0.05 (-0.46)	0.02 (0.13)	-0.04 (-0.37)	-0.12 (-1.38)	-0.06 (-0.63)
SZ: Schwyz	0.05 (0.50)	0.29 (1.63)	-0.03 (-0.25)	-0.05 (-0.43)	0.05 (0.31)	-0.04 (-0.33)		0.14 (1.09)	0.10 (0.86)	-0.07 (-0.48)	0.02 (0.18)	0.08 (0.46)	-0.09 (-0.77)	-0.09 (-0.68)	0.08 (0.66)	0.15 (1.17)	0.10 (0.94)	0.01 (0.14)	0.08 (0.75)
TG: Thurgau	0.09 (1.18)	0.33* (2.01)	0.01 (0.11)	-0.01 (-0.14)	0.09 (0.61)		0.04 (0.33)	0.17 (1.69)	0.14 (1.52)	-0.03 (-0.25)	0.06 (0.72)	0.11 (0.77)	-0.06 (-0.56)	-0.06 (-0.47)	0.12 (1.15)	0.19 (1.72)	0.14 (1.66)	0.05 (0.70)	0.12 (1.48)
TI: Ticino	0.00 (0.02)	0.24 (1.24)	-0.08 (-0.57)	-0.10 (-0.75)		-0.09 (-0.61)	-0.05 (-0.31)	0.09 (0.59)	0.05 (0.36)	-0.12 (-0.74)	-0.03 (-0.22)	0.03 (0.16)	-0.14 (-1.10)	-0.14 (-0.96)	0.03 (0.24)	0.10 (0.67)	0.05 (0.38)	-0.03 (-0.27)	0.03 (0.23)
VD: Vaud	0.10 (1.19)	0.34* (2.02)	0.02 (0.34)		0.10 (0.75)	0.01 (0.14)	0.05 (0.43)	0.19 (1.70)	0.15 (1.51)	-0.02 (-0.19)	0.07 (0.79)	0.13 (1.01)	-0.04 (-0.41)	-0.04 (-0.45)	0.13 (1.71)	0.20 (1.75)	0.15 (1.62)	0.07 (0.83)	0.13 (1.47)
VS: Valais	0.08 (1.03)	0.32 (1.93)		-0.02 (-0.34)	0.08 (0.57)	-0.01 (-0.11)	0.03 (0.25)	0.16 (1.60)	0.13 (1.39)	-0.04 (-0.39)	0.05 (0.59)	0.10 (0.81)	-0.07 (-0.67)	-0.06 (-0.67)	0.11 (1.37)	0.18 (1.62)	0.13 (1.50)	0.04 (0.59)	0.11 (1.35)
ZG: Zug	-0.24 (-1.54)		-0.32 (-1.93)	-0.34* (-2.02)	-0.24 (-1.24)	-0.33* (-2.01)	-0.29 (-1.63)	-0.15 (-0.91)	-0.19 (-1.17)	-0.36 (-1.89)	-0.27 (-1.72)	-0.22 (-1.06)	-0.39* (-2.26)	-0.38* (-2.12)	-0.21 (-1.19)	-0.14 (-0.79)	-0.19 (-1.19)	-0.28 (-1.77)	-0.21 (-1.34)
ZH: Zurich		0.24 (1.54)	-0.08 (-1.03)	-0.10 (-1.19)	-0.00 (-0.02)	-0.09 (-1.18)	-0.05 (-0.50)	0.09 (0.95)	0.05 (0.62)	-0.12 (-0.98)	-0.03 (-0.51)	0.02 (0.18)	-0.15 (-1.65)	-0.14 (-1.34)	0.03 (0.34)	0.10 (1.03)	0.05 (0.75)	-0.04 (-0.67)	0.03 (0.45)
	ZH	ZG	VS	VD	TI	TG	SZ	SO	SG	NE	LU	JU	GR	GE	FR	BS	BL	BE	AG

**Notes:** Each column reports the marginal effect of the canton of residence (row) on the financial hardship compared to the reference canton (column)

For example, the first column reports the financial hardship of each canton compared to the canton of Zurich (ZH)

*p < 0.05, **p < 0.01, ***p < 0.001. Robust standard errors in parenthesis

After controlling for several socio-demographic characteristics, the results show that all cantons have a higher probability of financial hardship compared with the canton of Zug (ZG). Nevertheless, this result was statistically significant only for the cantons of Geneva (GE), Graubünden (GR), Thurgau (TG) and Vaud (VD). More specifically, people living in the canton of Graubünden (GR) have a 38.6% higher probability of financial hardship compared with people living in Zug (ZG). This result is followed by the cantons of Geneva (GE) with 38.5%, Neuchâtel (NE) with 36.4%, and Vaud (VD) with 34.3%.

**Fig.1:**
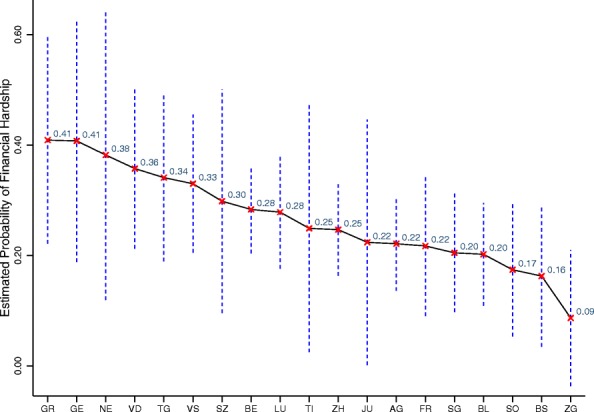
Probability of financial hardship of people with SCI by canton of residence

**Fig. 2:**
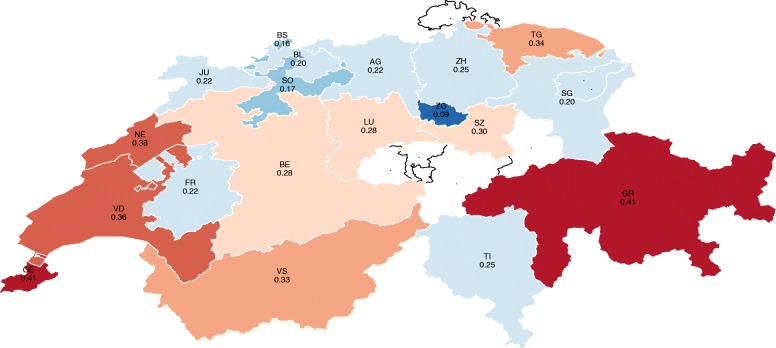
Probability of financial hardship of people with SCI by canton of residence

**Fig. 3:**
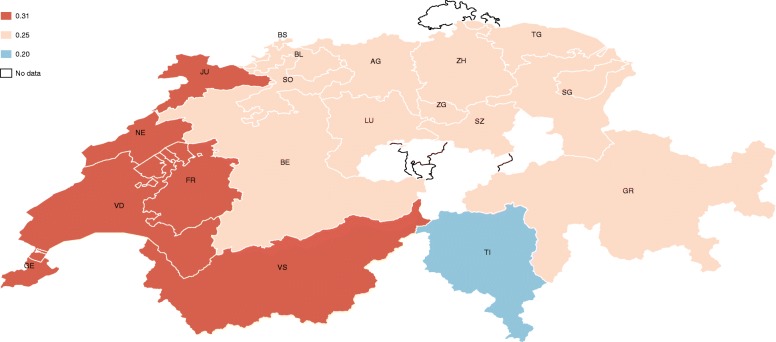
Probability of financial hardship of people with SCI by language region

For ease of comparison, the estimates are displayed in descending order in Fig. 1 and in a colored map in Fig. 2. The map shows there exists high variability in the results, where the probability of financial hardship ranges from 9% for households living in Zug (ZG) to 41% for households living in Graubünden (GR) and Geneva (GE), which is almost five times higher. The cantons of Neuchâtel (NE) and Vaud (VD) report probabilities of around 38 and 36%, respectively. Thurgau (TG) and Valais (VS) show probabilities of around 34%. Central Switzerland, mainly formed of German-speaking cantons, shows probabilities of below 30%. In the Italian region, Ticino shows a financial hardship probability of 25%. On average, cantons in the French-speaking region face a significantly higher probability of financial hardship compared with the German and Italian regions (Fig. 3).

Besides the canton of residence, there exist some other determinants of financial hardship that are worthy of mention. As expected, age displays an inverted U-shape pattern; getting older significantly increases the probability of financial hardship up to a limit. Once a person has enough education and experience, it is expected that they will have a better financial situation. These results complement the occupational situation; being unemployed seems to be the main driver of financial hardship in the sample.

Having children still living in the household increases the probability of financial hardship by 8.5%, which reflects the additional resources, in terms of time and money, children require. A higher household income and a better perception of quality of health significantly decrease the probability of financial hardship by 11.2 and 8.3%, respectively. Finally, SCI resulting from a traumatic event reduces by about 8% the probability of financial hardship, which reflects that people covered by accident insurance are better off in the sample. The remaining covariates show no significant results.

### Robustness check

**Fig. 4:**
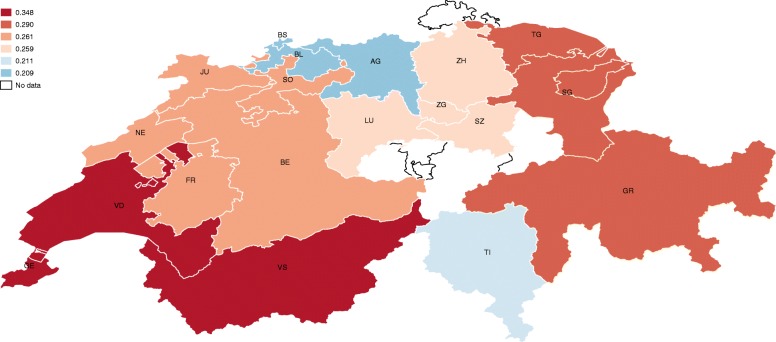
Probability of financial hardship of people with SCI by statistical region

Table 5 reports the results of Eq. (1) by using different definitions of the place of residence. The estimates compare language region, statistical region and cantons, results mapped in Fig. 3 and Fig. 4. As it is possible to see, the results are quite robust. Among the covariates, there are no marked differences between the three specifications; the occupational situation, the health status, the household composition, and income level are the main determinants of financial hardship in the sample.

**Table 5:** Robust Check: Estimated Marginal Effects by Place of Residence

	Language region	Statistical region	Canton
Personal/family characteristics:			
sex: male	0.055	0.050	0.050
	(0.032)	(0.033)	(0.034)
age	0.016*	0.016^*^	0.016^*^
	(0.007)	(0.007)	(0.007)
age^b^	−0.000*	− 0.000^*^	− 0.000^*^
	(0.000)	(0.000)	(0.000)
nationality: Swiss	−0.027	− 0.031	− 0.035
	(0.053)	(0.055)	(0.055)
health quality	−0.075^***^	−0.075***	− 0.078***
	(0.014)	(0.014)	(0.015)
SCI type: paraplegia	0.026	0.014	0.017
	(0.030)	(0.031)	(0.031)
SCI cause: traumatic	−0.089**	−0.095^**^	− 0.094^**^
	(0.034)	(0.035)	(0.035)
has a partner	0.055	0.047	0.042
	(0.031)	(0.032)	(0.032)
children in the household	0.080^*^	0.079^*^	0.085^*^
	(0.035)	(0.037)	(0.037)
Spoken language:^a^			
German	−0.012	0.017	0.071
	(0.115)	(0.114)	(0.113)
French	−0.108	− 0.098	− 0.058
	(0.119)	(0.117)	(0.117)
Education level:^b^			
vocational	0.036	0.018	0.016
	(0.054)	(0.057)	(0.057)
secondary	0.013	−0.004	−0.011
	(0.053)	(0.056)	(0.056)
university	0.099	0.081	0.070
	(0.061)	(0.063)	(0.064)
Occupational situation:			
full-time job	0.049	0.097	0.076
	(0.093)	(0.096)	(0.090)
part-time job	0.090	0.136	0.120
	(0.094)	(0.097)	(0.091)
in education	−0.003	0.016	0.010
	(0.074)	(0.076)	(0.078)
unpaid work	0.028	0.043	0.023
	(0.083)	(0.089)	(0.090)
unemployed	0.197^**^	0.205^*^	0.200*
	(0.071)	(0.080)	(0.079)
homemaker	−0.072	−0.084	−0.073
	(0.041)	(0.043)	(0.044)
invalidity pension	−0.012	− 0.048	− 0.028
	(0.093)	(0.096)	(0.090)
retired	0.002	−0.017	−0.026
	(0.051)	(0.053)	(0.054)
other activity	0.149^*^	0.156^*^	0.152^*^
	(0.066)	(0.068)	(0.065)
household income	−0.110***	−0.110***	− 0.110^***^
	(0.010)	(0.010)	(0.010)
Place of residence:			
French-speaking region	0.057		
	(0.057)		
Italian-speaking region	−0.059		
	(0.130)		
Lake Geneva		0.138*	
		(0.060)	
Espace Mittelland		0.057	
		(0.046)	
Zurich		0.048	
		(0.052)	
Eastern Switzerland		0.085	
		(0.052)	
Central Switzerland		0.055	
		(0.053)	
Ticino		0.003	
		(0.133)	
AG: Aargau			0.213
			(0.158)
BE: Bern			0.277
			(0.156)
BL: Basel-Landschaft			0.191
			(0.161)
BS: Basel-Stadt			0.139
			(0.175)
FR: Fribourg			0.208
			(0.175)
GE: Geneva			0.385^*^
			(0.182)
GR: Graubunden			0.386^*^
			(0.171)
JU: Jura			0.216
			(0.203)
LU: Lucerne			0.272
			(0.159)
NE: Neuchatel			0.364
			(0.192)
SG: St. Gallen			0.194
			(0.165)
SO: Solothurn			0.155
			(0.171)
SZ: Schwyz			0.291
			(0.179)
TG: Thurgau			0.329^*^
			(0.164)
TI: Ticino			0.243
			(0.196)
VD: Vaud			0.343^*^
			(0.170)
VS: Valais			0.320
			(0.166)
ZH: Zurich			0.241
			(0.156)
Observations	1300	1233	1233

Robust standard errors in parenthesis

For discrete variables, the marginal effects should be interpreted respect to the reference group:

^a^German is the reference group

^b^Compulsory education is the reference group

*p < 0.05, **p < 0.01, ***p < 0.001

However, when comparing the results of the geographical variables, it is possible to see that significant effects only appear as the variables are disaggregated. This result supports the main conclusion of this study, which is that financial hardship for people in need of long-term care is not entirely related to the health system, or to specific characteristics of the household, but also to the place of residence.

## Discussion

The aim of this paper was to determine whether financial hardship varies by canton of residence for people with SCI in Switzerland. As financial hardship has been shown to have a negative effect on health outcomes, its spatial variation implies that some people face a significant disadvantage because of their place of residence.

The results show that the probability of financial hardship varies widely across the country, ranging from 9% in the canton of Zug, to 41% in the cantons of Graubünden and Geneva—five times more. Among the 19 cantons analyzed (out of 26 cantons), people living in the cantons of Graubünden, Geneva, Vaud and Thurgau show significant results, with the probability of financial hardship between 30 and 40%. On average, people living in the Swiss-French speaking region experience a higher probability of financial hardship compared with the other regions, of which the cantons located in the Lake Geneva area are the most affected.

For people in need of long-term care, and following previous literature in the topic, it is possible to think about several potential sources of inequality that could explain the marked differences in financial hardship among cantons. Some more related to the health system, and others related to the geographical context of the cantons.

### Health system

#### Availability and accessibility

The availability and accessibility of health providers is not an issue in Switzerland. The country has a privileged position with respect to the number of hospitals and specialized clinics in the country compared to other OECD countries. Even if there exist some differences, patients are free to move between cantons to seek treatment. In fact, evidence shows that people with SCI are more likely to seek treatment outside their residential canton [32], behavior that reflects their preferences for specialized care.

#### Cost of health services (premiums)

Insurance premiums are set by the place of residence, irrespective of income; this puts more pressure on the financial situation of households at low-income levels [24]. Annual premiums reflect the health care costs of the region, which vary enormously between cantons. Big urban areas have the highest premiums, with Geneva at the top at 6550 CHF [33].

With prices varying from canton to canton, the out-of-pocket expenditure on health services also depends on the place of residence. On average, households experience an out-of-pocket expenditure close to 26% of total health spending, which is a rate significantly higher when compared with other OECD countries [11, 12]. It is estimated that 40% of the total budget for long-term care is financed by the public system and social insurance, and 60% by households in Switzerland [34].

#### Financial support

Due to the marked differences between cantons and the rising costs of health care, each canton has a budget to subsidize the health care costs of low-income families [12]. Besides contributing to the costs of care in nursing homes, the cantons provide financial support through invalidity pensions and supplementary insurances. People with severe, moderate or mild invalidity can benefit from invalidity insurance. The amount of benefits depends on the level of disability and the place of care. As for supplementary benefits, elderly, and disabled people who are unable to finance their living expenses are eligible to receive financial support.

Nevertheless, even though very generous, the financial support is administered by every canton, which creates different treatments for similar families. There is no regulation at the federal level about eligibility criteria or requirements to access funds. Instead, each canton designs and implements their own rules regarding social benefits. In most cases, the application for financial support can be time-consuming and require an in-depth knowledge of the system, thus creating important barriers that could keep some households out of the system.

### Geographical context

#### Socio-economic conditions

A potential explanation for the geographical differences in financial hardship might be related to the economic environment in each canton. Nevertheless, if we compare the probability of financial hardship among the richest cantons, or the poorest, there is no general rule. In fact, rich cantons such as Basel, Geneva, Zurich and Zug report substantially different probabilities of financial hardship. The same is true in smaller and more rural cantons.

Nevertheless, even when people could have more trouble finding a job in their place of residence due to the economic environment, mobility is quite frequent. In fact, 7 out 10 people work outside their place of residence [33].

#### Taxation

**Fig. 5:**
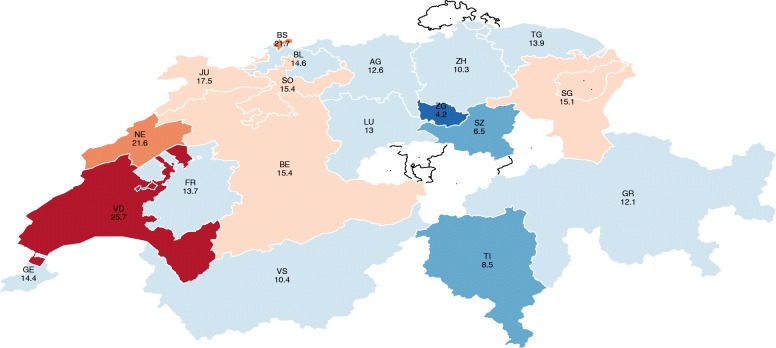
Tax burden by canton of residence (2013)

Taxation is an important element in the autonomous political system of Switzerland. Depending on the place of residence, the family composition, and income level, households face significantly different tax rates. For example, for a gross annual income of between CHF 60000 and 80,000, a married couple with no children pays 4.2% in Zug compared with 25.7% in Vaud—over five times more (Fig. 5) [35]. As with the health system, families can apply for financial support through deductions, but the criteria which determine a deduction, and their conditions and implementation, are regulated by the cantonal administration.

#### Cultural differences

Finally, the marked and persistent differences between language regions suggest that cultural factors have an important influence on the financial situation of people. This result compliments related literature, which shows that the behavior of Swiss-French and Swiss-Italian residents has a direct impact on health care costs, compared with their counterparts in the Swiss-German region.

## Limitations

The number of participants within cantons poses some limitations for this study. Many cantons have few participants, which causes the estimation to be less precise. In addition, due to privacy issues, the estimation excluded cantons with less than 15 participants, meaning that the remainder may have been dominated by bigger and richer cantons.

In addition, the sample is composed of people with SCI mostly caused by a traumatic event (78.4%). This implies that most people are covered by accident insurance and not by regular health insurance. In general, accident insurance comprises a more generous package than health insurance; there is no deductible and people receive higher compensation. Therefore, people in the sample are less likely to suffer from financial hardship compared with other long-term conditions; this may cause some underestimation of results.

Finally, even though the cross-section dataset used in this study serves as a good framework from which to analyze geographical differences, it has some shortcomings. For people with SCI, the time between medical discharge and job reintegration could be a determinant of their future financial situation, whereas people who stay unemployed for longer periods are less likely to find a job. In addition, people with lower education tend to be more affected because their jobs require more physical effort. Having a SCI is a condition that limits the physical capabilities of a person; people with fewer skills will face more difficulties when searching for a job. In many cases, they need to acquire more education and training in order to switch to another economic activity. A longitudinal analysis would improve this study by making possible a comparison between subjects before and after the injury took place, and establishing the time people need to go back to work.

## Conclusion

The place of residence has a significant impact on family budget. In Switzerland, depending on the canton of residence, families face different health care costs, taxes, and economic environment. This situation has raised important inequalities within the country, especially for those households that have a family member requiring constant care. Even though the cantonal administrations have tried to tackle this problem through financial support, significant differences persist within the country, putting some families at a disadvantage.

## Abbreviations

SCI: Spinal cord injury
SwiSCI: Swiss Spinal Cord Injury Cohort Study
